# Comparison of antimicrobial resistant genes in chicken gut microbiome grown on organic and conventional diet

**DOI:** 10.1016/j.vas.2016.07.001

**Published:** 2016-08-12

**Authors:** Narasimha V. Hegde, Subhashinie Kariyawasam, Chitrita DebRoy

**Affiliations:** Department of Veterinary and Biomedical Sciences, E. coli Reference Center, The Pennsylvania State University, University Park, PA, United States

**Keywords:** Chicken, Gut microbiome, Antimicrobial resistance genes, Metagenomic library, Phylotypes

## Abstract

Antibiotics are widely used in chicken production for therapeutic purposes, disease prevention and growth promotion, and this may select for drug resistant microorganisms known to spread to humans through consumption of contaminated food. Raising chickens on an organic feed regimen, without the use of antibiotics, is increasingly popular with the consumers. In order to determine the effects of diet regimen on antibiotic resistant genes in the gut microbiome, we analyzed the phylotypes and identified the antimicrobial resistant genes in chicken, grown under conventional and organic dietary regimens. Phylotypes were analyzed from DNA extracted from fecal samples from chickens grown under these dietary conditions. While gut microbiota of chicken raised in both conventional and organic diet exhibited the presence of DNA from members of *Proteobacteria* and *Bacteroidetes,* organic diet favored the growth of members of *Fusobacteria*. Antimicrobial resistance genes were identified from metagenomic libraries following cloning and sequencing of DNA fragments from fecal samples and selecting for the resistant clones (*n*=340) on media containing different concentrations of eight antibiotics. The antimicrobial resistant genes exhibited diversity in their host distribution among the microbial population and expressed more in samples from chicken grown on a conventional diet at higher concentrations of certain antimicrobials than samples from chicken grown on organic diet. Further studies will elucidate if this phenomena is widespread and whether the antimicrobial resistance is indeed modulated by diet. This may potentially assist in defining strategies for intervention to reduce the prevalence and dissemination of antibiotic resistance genes in the production environment.

## Introduction

Administration of antimicrobials to chickens at therapeutic and sub-therapeutic levels has been an integral part of poultry production in the US. Antimicrobials have been widely used in the poultry industry, for therapeutic purposes, disease prevention and growth promotion. It is now accepted that use of antibiotics in farms selects for drug resistant organisms which can then spread from farm to humans through consumption of contaminated food (Hawkey, 2008). Research on antimicrobial resistance in foodborne pathogens have demonstrated that use of antimicrobials in agriculture can result in drug resistant bacteria isolated from humans (Angulo et al., 2004, Hawkey, 2008, Hawser, 2012, USDA National Organic Program, 2008).

To avoid consuming antimicrobial resistant bacteria, United Stated Department of Agriculture (USDA) established the National Organic Program that has grown by almost 20% annually in the U.S. since 1990 (http://www.apsnet.org/publications/apsnetfeatures/Pages/Organics.aspx). Organic poultry production focuses on poultry health, good environmental practices, production quality and reduced use of antibiotics, hormones or animal byproducts in feed as mandated by USDA. (http://www.usda.gov/wps/portal/usda/usdahome?navid=organic-agriculture). Conventional poultry production that accounts for about 95% of poultry grown in the U.S. (MacDonald, 2008) focuses on reducing costs and maximizing production through utilizing rapidly growing species that are sometimes fed antimicrobials and dietary supplements.

Diet plays a major role in modulating gut microflora and it is widely accepted that antibiotics in the diet can provide selective pressure on microbial community that may facilitate persistence and the transfer of resistance determinants between bacterial species (Andersson & Hughes, 2012; Wright, 2010) leading to the emergence of drug resistant bacteria. It is becoming evident that many non-pathogenic commensal bacterial species play a role in the development of antibiotic resistance and further transmission of resistance determinants (Marshall et al., 2009, Witte, 2000).

While most of the research on antimicrobial resistance has focused on foodborne pathogens, which are a fraction of microbiota population in the gut, little is known on the distributions and expression of antibiotic resistance genes in the general microbiota of chicken gut that may provide valuable information on the emergence of drug resistance. Metagenomic functional selection of antibiotic resistance genes assists in better understanding of genetic exchanges between diverse microbial species (de la Cruz and Davies, 2000, Lester et al., 2006, Shoemaker et al., 2001) and have also identified the existence of antimicrobial resistant genes in bacteria isolated from the environment which have not been exposed to antibiotics (D’Costa et al., 2011, Moore et al., 2013). Extensive research has been carried out to understand the mechanism of emergence of drug resistance in bacteria, and of antibiotic resistance reservoirs from diverse microbial communities (Andersson and Hughes, 2012, Danzeisen et al., 2011, Durso et al., 2011) and to trace the origin of new and emerging antimicrobial resistance in pathogenic bacteria (Angela et al., 2011, Solberg et al., 2006). In this investigation, we analyzed the phylotypes, and prepared a metagenomics library from fecal samples of chicken grown on organic and conventional diets to identify antibiotic resistance genes and their expression in the presence of a gradient of eight antimicrobials. Using a small set of samples, as proof of principle, we found that the diet does plays a role in modulating the antimicrobial resistance in the chicken gut. The results exhibited that at higher concentrations of certain antimicrobials a greater number of resistance genes were expressed in samples associated with conventional diet than organic. To the author's knowledge, the functional genomics of antimicrobial resistance genes in the presence of a gradient of antimicrobials under organic and conventional dietary regimens in chicken gut microbiome have not been studied before. While this study has been conducted with a limited number of samples, further research on a larger scale may lead to understanding how the expression of the antimicrobial resistance genes differ in microbiomes under many different dietary conditions.

## Material and methods

### Diet regimens for chickens

Twenty commercial layer chickens (90-day-old, Brown Leghorns) grown under two dietary regimens, organic (OD) (*n*=10), and conventional (CD) (*n*=10) were raised in a farm following USDA guidelines (USDA National Organic Program, 2008). The chickens on the conventional diet were treated with chlortetracycline for the first 9 days at 400 g/ton of feed as treatment for presumptive infection by bacteria like *E. coli*. The chickens on the organic diet regimen were not exposed to antibiotics. The feeds were otherwise similar in nature in the conventional and organic diet. Fresh fecal matter from 10 chickens under each dietary regimen were collected and shipped to the laboratory from the farm.

### Phylotype analysis

DNA was extracted from fresh fecal samples from each group of 10 chickens (5 g feces from each bird) using the PowerMax fecal DNA Isolation Kit (MoBio Laboratories Inc. Carlsbad, CA) according to the manufacturer's protocol. DNA isolated from fecal samples (*n*=10) from each dietary group were pooled. Concentration of total genomic DNA isolated from each group was estimated using Qubit® Fluorometer (Invitrogen, Molecular Probes, Eugene, OR). Pooled DNA (0.1 µg) from each group was used for amplification of 16S ribosomal RNA genes (rRNA) using universal bacterial 16S primers (F-AGAGTTTGATCTGGCTCAG and R-CCCCGTCAATTCTTTGAGTTT). PCR mixtures (50 μl) contained PCR buffer, 0.2 mM of each of dNTP, 0.4 mM each primer (Integrated DNA Technology, Coralville, IA), 2.5 U of FastStart High Fidelity *Taq* polymerase, and 50 ng template DNA. PCR was performed with thermocycler^d^ programmed to perform 95 °C for 2 min, followed by 30 cycles of 95 °C for 30 s, 60 °C for 30 s, 72 °C for 30 s followed by final extension at and 72 °C for 7 min. PCR products were visualized by agarose gel electrophoresis and DNA band excised, purified from the gels by Qiaquick columns (Qiagen, Valencia, CA) PCR products were sequenced on a 454 Genome Sequencer FLX at Genomics Core facility at the Pennsylvania State University. The samples were barcoded, taxonomic assignments of the sequenced 16S rRNA gene were made using the Ribosomal Database Project (RDP) Web tools (Roche Diagnostics, Indianapolis, IN).

Following sequencing, the barcodes were sorted, the 16S rRNA sequences were screened for quality. The sequences that did not match the primer sequences in the beginning and end of the reads were eliminated to minimize errors. RDP database was used for taxonomic grouping with a boot strap cut off of 80% for statistical analysis (Cole et al., 2014). To determine the operational taxonomic unit (OUT) Mothur was used (Schloss et al., 2009) with a definition at a similarity cutoff of 95%.

### Library construction

Pooled DNA (2 µg) from each dietary groups (OD or CD) were partially digested with *Sau*3AI ( New England BioLab, Boston, MA) size fractionated using agarose gel electrophoresis and 1500–2500 bp fragments were purified using QIAquick Gel Extraction Kit Eppendorf, Germany.

Plasmid vector (Roche Diagnostics, Indianapolis, IN) pAcGFP1–1 was digested with *BamH*I dephosphorylated using Antarctic Phosphatase (New England BioLab, Boston, MA) and ligated to plasmid vector pAcGFP1–1 using the Fast-Link DNA Ligation Kit (Epicentre, Madison, WI) following the protocol provided by the manufacturer.

Cloned DNA (3 μL) was used for transforming 40 μL of chemically competent *E. coli* (NEB 10-beta) (New England BioLab, Boston, MA) following the high efficiency transformation protocol provided by the manufacturer. For each library, forty transformation reactions were pooled and libraries were titered by plating out 25 μL of recovered cells onto LB agar plates containing 50 μg/mL kanamycin. For each library, insert size distribution was estimated by gel electrophoresis of PCR products obtained by amplifying the insert using primers flanking the *Bam*HI site of the multiple cloning site of the pAcGFP1–1 vector. PCR mixtures (20 μl) contained PCR buffer, 0.2 mM of each of dNTP, 0.4 mM each primer (ACG-F: 5′ CAG TCG ACG GTA CCG CGG GCC 3′ and ACG-R: 5′CAC CAT GGT GGC GAC CGG3′), 2.5 U of FastStart High Fidelity *Taq* polymerase, and 50 ng template DNA. PCR was performed with thermocycler^d^ programmed for 95 °C for 2 min, followed by 30 cycles of 95 °C for 30 s, 60 °C for 30 s, 72 °C for 90 s followed by final extension at and 72 °C for 7 min.

### Functional selection of antibiotic resistant clones from metagenomic libraries

The screening protocol described (Sommer, Dantas & Church, 2009) antibiotic resistant clones from each library were selected by plating 1 mL of cultures corresponding to 1.5–2×10^6^ CFU on Luria Bertani (LB) agar plates containing kanamycin (50 μg/mL) and one of eight antibiotics of interest at different concentrations (4–32 µg/ml). The antibiotics used belonged to different groups. Amoxicillin and penicillin (β-lactams), chloramphenicol and florophenicols (amphenols), gentamycin and spectinomycin (aminoglycosides) and tetracycline and oxytetracycline (tetracycline group) were used. The plates were incubated at 37 °C for 16 h. Resistant colonies (*n*=340) from both OD and CD libraries were picked for further analysis. As a negative control, for each antibiotic, NEB 10-beta *E. coli* cells containing pAcGFP1-1 vector was plated on LB agar plates containing kanamycin (50 μg/mL) and one of the antibiotics, at the same concentrations as that used for selection for functional screening. A total of 340 inserts conferring resistance were sequenced by using the primers used for the library construction.

### Sequencing and analysis of metagenomic inserts

Selected clones were sequenced using Sanger sequencing. A total of 340 clones were sequenced bi-directionally using the primers ACG-F: 5′ CAG TCG ACG GTA CCG CGG GCC 3′ and ACG-R: 5′ CAC CAT GGT GGC GAC CGG 3′. Amplified targets were analyzed using BLAST search and the sequence similarity (>96%, nucleotide level) of the resistance genes were compared using GenBank (http://www.ncbi.nlm.nih.gov).

## Results and discussion

### Phylotype analysis

Analyses of a total of about 800,000 amplified DNA fragments were conducted using the Ribosomal Database Project (RDP) (http://rdp.cme.msu.edu) that classified the DNA into diverse phyla, class, order, and genus. The bacterial phyla found in fecal samples from chickens on organic diet were *Firmicutes* (34.4%), *Fusobacteria* (40.5%), *Proteobacteria* (22.4%), *Bacteroidetes* (0.4%), *Actinobacteria* (0.3%), and unclassified (2%) while fecal samples from chickens on conventional diet exhibited the presence of bacterial phyla, *Firmicutes* (31.4%), *Fusobacteria* (12.1%), *Proteobacteria* (38.1%), *Bacteroidetes* (10.3%), *Actinobacteria* (1.3%), and unclassified (6.6%).

The predominant bacteria in samples from organic diet were *Clostridium* (38.5%), *Escherichia coli* (16.9%) *Lactobacillus* (12.3%) and *Bacteriodes* (0.4%). The percentages of bacteria in samples from conventional diet group were *Escherichia coli* (28%)*, Clostridium* (11.4%), *Bacteriodes* (10.3%) and *Lactobacillus* (6.8%). It was clear that certain bacterial species seem to thrive better in organic diet such as *Clostridium* and *Lactobacillus* and others in conventional diet such as *E. coli* and *Bacteriodes*. *Lactobacillus* were more abundant followed by *Clostridium* and *Bacteriodes* in small intestines in younger chicken grown in conventional diet (Mead & Adams, 1975). In older chickens, *Salmonella*, *Campylobacter* and *E. coli* were present (Zhou et al., 2012, Amit-Romach et al., 2004, Dumonceaux et al., 2006).

### Functional screening and identification of antimicrobial resistance genes

The metagenomic library of the DNA cloned from fecal samples of chicken grown in organic diet or conventional diet each comprised of 4.5–5×10^8^ clones. The mean insert size of the cloned DNA in the metagenomic library of samples from organic diet (OD library) and conventional diet (CD library) were 1.9 kb and 1.8 kb respectively. For functional selection of antimicrobial resistant genes, each library was screened for 8 antimicrobials, at 3 different concentrations (4, 8 and 16 µg/mL), with the exception of penicillin (32 µg/mL) and spectinomycin (64 µg/mL) where only one concentration was used for functional selection. Resistant clones from each selection event were sequenced and genes associated with the resistance for each class of antimicrobials were identified (Table 1). The results allowed understanding of the expression of antimicrobial resistance genes under higher selective pressure.

**Table 1:** Antimicrobial resistance genes identified using metagenomic functional selections of chicken gut microbiomes.

Antibiotic (conc.) (number of clones)	**Conventional Diet**					**Organic Diet**			
	**Top hit**		DNA % ID	Nucleotide Position (Gene length)	Gene	**Top hit**	DNA % ID	Nucleotide Position (Gene length)	Gene
	Bacteria [GB ID] (number of clones)					Bacteria [GB ID] (number of clones)			
**β lactam**									
Amoxicillin (4 µg/mL) (*n*=10)	*Escherichia coli* [CP004009.1] (*n*=6)		99	367564–368697 (1133)	*ampC* (β lactamase)	*Escherichia coli* [AP010960.1](n=10)	98	5154506–5155639 (1133)	*ampC* (d-alanine carboxypeptidase)
	*Escherichia coli* [AP010960.1] (*n*=4)		98	5154506–5155639 (1133)	*ampC* (d-alanine carboxypeptidase)				
Amoxicillin (8 µg/mL) (*n*=10)	*Escherichia coli* [AP010960.1] (*n*=8)		98	5154506–5155639 (1133)	*ampC* (d-alanine carboxypeptidase)	*Escherichia coli* [AP010960.1] (n=10)	98	5154506–5155639 (1133)	*ampC* (d-alanine carboxypeptidase)
	*Enterobacter asburiae* [EU427302.2](*n*=2)		98	1476–2618 (1142)	β lactamase ACT 3’				
Amoxycillin (16 µg/mL) (*n*=10)	*Escherichia coli* [CP002967.1](*n*=10)		99	4631143–4633212 (2089)	*sugE* (membrane transporter) *ampC* (β lactamase)	*Escherichia coli* [AP010960.1] (n=10)	98	5154506–5155639 (1133)	*ampC* (d-alanine carboxypeptidase)
Penicillin (32 µg/mL) (*n*=10)	*Escherichia coli* [AP010958.1] (*n*=5)		99	5135487–5137556 (2069)	*sugE* (membrane transporter) *ampC* (β lactamase)	*Escherichia coli* [AP010960.1] (n=10)	98	5154506–5155639 (1133)	*ampC* (d-alanine carboxypeptidase)
	*Escherichia coli* [CP004009.1] (*n*=5)		98	367564–368697 (1133)	*ampC* (β lactamase)				
**Amphenols**									
Chloramphenicol (4 µg/mL) (*n*=10)	*Escherichia coli* [CP003301.1] (*n*=10)		99	3550043–3551275 (1232)	*mdfA* (multidrug efflux system translocase)	*Escherichia coli* [CP002291.1] (*n*=10)	96	1663943–1664581 (638)	*marA-marB* (multiple antibiotic resistance operon)
Chloramphenicol (8 µg/mL) (*n=*10)	*Escherichia coli* [AP010958.1] (*n*=4)		99	968295–969527 (1232)	*cmr* (multidrug efflux system)	None			
	*Escherichia coli* [CU928160.2] (*n*=6)		98	935786–937018 (1232)	*cmr* (multidrug efflux system)				
Chloramphenicol (16 µg/mL) (*n*=10)	*Escherichia coli* [AP010958.1] (*n*=5)		99	968295–969527 (1232)	*cmr* (multidrug efflux system)	None			
	*Escherichia coli* [CU928160.2] (*n*=6)		98	935786–937018 (1232)	*cmr* (multidrug efflux system)				
Florfenicol (4 µg/mL) (*n*=10)	*Escherichia coli* [CP003301.1] (*n=*10)		99	4494426–4493557 (869)	*creB-araC(*response regulator -transcription activator)	*Escherichia coli* [AP010958.1] (*n*=10)	96	5442103–5443656 (1553)	*rob-creA* (transcription activator)
Florfenicol (8 µg/mL) (*n*=10)	*Escherichia coli* [CP002967.1] (*n=*10)		99	2240178–2241239 (1061)	UDP-Glucose-4-epimerase Glycosyl transferase	*Escherichia coli* [AP009240.1] (*n*=10)	96	2212192–2211261 (931)	ABC transporter
Florfenicol (16 µg/mL) (*n*=10)	None					None			
**Aminoglycoside**									
Gentamicin (4 µg /mL) (*n*=10) (8 µg/mL) (*n*=10)	*Campylobacter jejuni* [AY701528.1] (*n*=12)		98	9696–10589 (893)	*aacA/aphD* (bifunctional amino- glycoside N-acetyl/phospho transferase )	*Staphylococcus aureus* [CP005288.1] (*n*=13)	96	2265687–2267126 (1439)	*aacA/aphD (*bifunctional amino- glycoside N-acetyl/phospho transferase )
	*Staphylococcus aureus* [HF569094.1] (*n*=8)		99	36923–38362 (1439)	*aacA/aphD (*bifunctional amino- glycoside N-acetyl/phospho transferase )	*Enterococcus faecium* [AB206333.1] (n=7)	96	62171–63610 (1439)	*aacA/aphD (*bifunctional amino- glycoside N-acetyl/phospho transferase )
Gentamicin (16 µg/mL) (*n*=10)	*Staphylococcus epidermidis* [JX910899.1] (*n*=8)		98	1303–2742 (1439)	*aacA/aphD* (bifunctional amino- glycoside N-acetyl/phospho transferase )	None			
	*Streptococcus faecalis* [M13771.1] (n=2)		98	304–1743 (1439)	*aacA/aphD* (bifunctional amino- glycoside N-acetyl/phospho transferase )				
Spectinomycin (64 µg/mL) (*n*=10)	*Bacteroides uniformis* [AY345595.1] *(n=*3)		95	38057–38677 (620)	acetyltransferase gram positive like	None			
	*Salmonella enterica* [AB576781.1] (*n*=4)		99	17352–18362 (1010)	spectinomycin/streptomycin adenyltransferase				
	*Clostridium cellulolyticum* [CP001348.1] (*n*=3)		98	3366196–3367416 (1220)	N-acetyltransferase				
**Tetracycline**									
Oxytetracycline (4 µg /mL) (*n*=10) (8 µg/mL) (*n*=10)	*Corynebacterium glutamicum* [AF121000.1] (*n*=12)		99	13133–13732 (599)	*tetR* (tetracycline repressor)	*Mennheimia haemolytica* [CP005383.1] (*n*=20)	99	2178268–2178891 (623)	*tetR* (tetracycline repressor)
	*Corynebacterium glutamicum* [AF121000.1] (*n*=8)		99	11880–13034 (1154)	*tetA*(tetracycline resistance)				
Oxytetracycline 16 µg/mL) (*n*=10)	Uncultured bacterium [FJ012881.1] (*n*=10)		99	40470–41630 (1160)	*tetX*(tetracycline resistance)	Uncultured bacterium [KC734562.1] (*n*=10)	99	3601–5520 (1919)	*tetR-tetH*(tetracycline resistance)
Tetracycline (4 µg /mL) (*n*=10) (8 µg/mL) (*n*=10)	*Corynebacterium glutamicum* [AF121000.1] (*n*=11)		99	13133–13732 (599)	*tetR* (tetracycline repressor)	*Enterococcus faecium* [HM636636.1] (*n*=11)	97	18678–20597 (1919)	*tetR-tetH*(tetracycline resistance)
	*Corynebacterium glutamicum* [AF121000.1] (*n*=9)			11880–13034 (1154)	*tetA*(tetracycline resistance)	*Streptococcus agalactiae [HF952106.1] (n=9)*	95	639169–641088 (1919)	*tetM* (tetracycline resistance)
Tetracycline 16 µg/mL) (*n*=10)	*Acinetobacter sp* [AY743590.1] (*n*=10)		99	42–1936 (1894)	*tetR- tetA*(tetracycliner resistance)	Uncultured bacterium [FJ012881.1] (*n*=10)	97	40470–41630 (1160)	*tetX*(tetracycline resistance)

Screening of both OD and CD libraries for antimicrobial resistance genes to beta-lactam class of antibiotics (amoxicillin, penicillin) exhibited the presence of chromosomally associated genes encoding for d-alanine carboxypeptidase, (*ampC)* associated with *E. coli*. Screening for resistance genes for higher concentration of amoxicillin (16 µg/mL) showed the presence of a membrane transporter (*sugE*) as well as beta lactamase associated with *E. coli* only, found in the CD library. The OD library did not exhibit any antimicrobial resistance genes at this concentration. It is known that β-lactamases (*ampC)* are normally encoded in the chromosome of many *Enterobacteriaceae* members and its activity is known to increase when they are mobilized into plasmids (Jacoby, 2009, Reisbig and Hanson, 2004).

Resistance determinants to the amphenicol class of antibiotics (chloramphenicol, florfenicol) were also found to be associated with *E. coli* species from both the libraries. At lower concentration of chloramphenicol (4 µg/mL), clones exhibited the presence of multidrug efflux system translocase (*mdfA*) in the CD library while clones with multiple antibiotic resistance operon *marA*-*marB* were selected from the OD library. With increase in the concentration of chloramphenicol in the selection medium (8–16 µg/mL) clones for multidrug efflux system (*cmr*) were obtained from the CD library while the OD library did not exhibit any genes for antimicrobial resistance at this concentration. In the presence of florfenicol (4–8 µg/mL) genes associated with *E. coli* carrying transcription activator genes (*creB*-*araC*) and UDP Glucose-4-epimerase glycosyl transferase were detected in the CD library whereas *E. coli* carrying transcription activator (*rob-creA*) and ABC transporter were observed in the OD library. None of the antimicrobial resistance genes were observed at higher concentrations of florfenicol in both the CD and OD libraries.

Antimicrobial resistance genes for aminoglycoside classes of antibiotics (gentamycin, spectinomycin) were functionally similar but differed in host distribution. The medium containing three different concentrations (4, 8, 16 µg/mL) of gentamycin selected clones for bifunctional aminoglycoside N-acetyl/phospho transferase (*aacA/aphD*). At concentrations (4–8 µg/mL) of gentamycin, *aacA/aphD* was found to be associated with *Campylobacter jejuni* and *Staphylococcus aureus* in the CD library whereas the same functional gene was found to be associated with *Staphylococcus aureus* and *Enterococcus faecium* in the OD library. At higher concentration of gentamycin (16 µg/mL) *aacA/aphD* gene was associated with *Staphylococcus epidermidis* and *Streptococcus faecalis* species in the CD library. No genes encoding for antimicrobial resistance was observed in the OD library at this concentration of gentamycin.

Different tetracycline resistant determinants associated with diverse bacterial species were observed when the tetracycline class of antibiotics (oxytetracycline, tetracycline) was used for screening the libraries. While tetracycline repressor *tetR* and tetracycline resistance *tetA* were associated with the *Corynebacterium glutamicum* at 4–8 µg/mL of oxytetracycline for the CD library, tetracycline repressor *tetR* was found to be associated with *Mennheimia haemolytica* for the OD library at the same concentration of oxytetracycline. Higher concentration of oxytetracycline (16 µg/mL) selected for the genes, *tetX* and *tetR-tetH* cassette, associated with unculturable bacteria from the CD and OD libraries respectively. Lower concentration of tetracycline (4–8 µg/mL) selected the genes containing *tetM* gene from *Enterococcus faecium* and *Streptococcus agalactiae* from the OD library and *tetA* and *tetR* associated with *Corynebacterium* from the CD library. At higher concentration (16 µg/mL) of tetracycline, *tetR-tetA* were selected that were associated with *Acinetobacter sp.* for CD and *tetX* was found to be associated with un-culturable bacteria for OD. Tetracycline resistance mechanisms have been attributed to efflux system (*tetM*), chemical inactivation (*tetX*), and repressor function (*tetR*) (Nelson and Levy, 2011, Ramos et al., 2005). In this study a lower concentration of tetracycline favored the selection of resistant determinants *tetR* and *tetM* while a higher concentration of tetracycline selected the clones containing *tetX* indicating that tetracycline concentration had a dependent functionality to resistance determinants. The results of the study have been summarised in Table 2.

**Table 2:** The antimicrobial resistance genes associated with samples from the organic and conventional diet regimens.

**Antibiotics**	**Conventional Diet**	**Organic diet**
Amoxicillin	*ampC, sugE*	*ampC*
Penicillin	*ampC, sugE*	*ampC*
Chloramphenicol	*mdfA, Cmr*	*marA-marB*
Florfenicol	*creB-araC, Glycosyl transferase*	*rob-creA, ABC transporter*
Gentamycin	*aacA/aphD*	*aacA/aphD*
Streptomycin	Adenyltransferase,	none
	N-acetyltransferase	
Oxytetracycline	*tetR, tetA, tetX*	*tetR, tetR-tetH*
Tetracycline	*tetR, tetA, tetR-tetA*	*tetR-tetH, tetM, tetX*

Antimicrobial resistance gene *ampC*, associated with *E. coli,* was found to be similar in fecal samples from chickens grown in both organic and conventional diet regimens (Table 1). The resistance genes were more prevalent in CD samples when grown in LB at higher concentration of antibiotics than from OD samples except for the common antibiotics, tetracycline and penicillin. No antimicrobial resistance genes were observed at higher concentrations of most of the antibiotics tested for OD. Detection of antibiotic resistant genes in chicken gut grown in organic diet supports the earlier postulated hypothesis that environmental bacteria may harbor diverse pool of antibiotic resistance regardless that environmental bacteria may harbour a diverse pool of antibiotic resistance regardless of potentially reduced exposure to antibiotics of exposure to antibiotics in organic farming (Aminov, 2009; Zhao et al., 2012). The results appear to support observations (D’Costa et al., 2011) that antibiotic resistance is an ancient natural phenomenon that does not depend on selective pressures due to clinical antibiotic usage.

Functional screening of antimicrobial resistance genes on antibiotic concentration gradients has provided some insight into the association of these genes with certain microbial species at sub-inhibitory concentrations. The study has also corroborated that relatively harmless commensal bacterial species may also be carriers of antimicrobial resistance, as has been suggested earlier (D’Costa et al., 2011). While the degree of expression of antibiotic resistance genes may have been biased because of cloning of the genes in *E. coli*, it may also be the case that these differ in the gut when other members of microbiome are in play. Further characterization of these antimicrobial resistance genes in the gut may allow better understanding of how antibiotic resistance emerges in the environment. In both organic and conventional diet samples *tetR* and *tetX* antimicrobial resistant genes for tetracycline were found to be associated with unidentified bacteria, and this may assist in improving our understanding of the relationship between species diversity and transfer of the genes between species. While sequences associated with most of the antibiotic resistance genes and their host species have been reported, there were some sequences that matched with uncultured bacteria. This disparity could be due to the fact that during submission of the sequences, sometimes the source is not mentioned if the sequences were deduced from metagenomics libraries or the complete sequence of the source bacteria is not available. Further research of gut microbiome under different diet regimens may assist in defining strategies for intervention to reduce the prevalence and dissemination of antibiotic resistance genes in the production environment.

## Declaration of conflicting interests

The authors declared no potential conflicts of interests with respect to the research, authorship, and/or publication of this article.
